# Predictors of pregnancy-associated venous thromboembolism: A case-control study

**DOI:** 10.3389/fcvm.2022.920089

**Published:** 2022-10-14

**Authors:** Mohammed A. Alsheef, Alhanouf M. Alabbad, Rowida A. Albassam, Rawan M. Alarfaj, Abdul Rehman Zia Zaidi, Ouhod A. Alarfaj, Mohsen Ayyash, Amani Abu-Shaheen

**Affiliations:** ^1^Department of Medical Specialties, King Fahad Medical City, Riyadh, Saudi Arabia; ^2^College of Pharmacy, Princess Nourah bint Abdulrahman University, Riyadh, Saudi Arabia; ^3^College of Medicine, Alfaisal University, Riyadh, Saudi Arabia; ^4^Pharmacy Service Administration, King Fahad Medical City, Riyadh, Saudi Arabia; ^5^School of Mathematical Sciences, University Sains Malaysia (USM), Gelugor, Malaysia; ^6^Scientific Writing Department, King Fahad Medical City, Riyadh, Saudi Arabia

**Keywords:** associated venous thromboembolism, pregnancy, VTE, deep vein thrombosis, DVT and PE

## Abstract

**Background:**

Venous thromboembolism (VTE), manifesting as pulmonary embolism (PE) or deep vein thrombosis (DVT), is the most common cause of morbidity and death during pregnancy and the postpartum period. We conducted this study to describe the predictors of pregnancy-associated VTE (DVT and PE).

**Methods:**

A case-control study was conducted at a tertiary care center in Riyadh. A total of 380 patients were included in this study, 180 of whom were diagnosed with pregnancy-associated thrombosis and 200 of them showed no VTE. Demographic data and data on risk factors of VTE were collected by reviewing the medical charts and the risk assessment tool of the Royal College of Obstetricians and Gynecologists, respectively. The main outcome measures were VTE, manifesting as PE or DVT.

**Results:**

The following factors were identified as the predictors of VTE through multivariate analysis: family history [Odds ratio (OR) = 50.47, 95% Confidence Interval (CI): 6.78–375.64, *P* < 0.0001)], thrombophilia (OR = 21.99, 95% CI: 2.83–170.63, *P* = 0.003), and presence of gross varicose veins (OR = 17.15, 95% CI: 3.93–74.87, *P* < 0.0001).

**Conclusions:**

The findings of this study showed that family history, thrombophilia, and the presence of gross varicose veins were risk factors for VTE, exceeding other transient risk factors. Hence, prophylaxis is highly recommended for those women who present with any of these factors.

## Introduction

Venous thromboembolism (VTE), which may manifest as pulmonary embolism (PE) or deep vein thrombosis (DVT), is a serious medical condition associated with significant morbidity and mortality and is expected to more than double in the next 40 years (1). It is predominantly a disease of old age and rarely occurs before late adolescence (2, 3). VTE incidence rates are slightly higher in women during their childbearing years, while in men it is generally higher after the age of 45 (4).

Age, body mass index (BMI), major surgery, hospitalization for acute illness, trauma, fracture, cancer, central vein catheterization or the presence of a trans-venous pacemaker, prior vein thrombosis, varicose veins, urinary tract infection, and a family history of venous thrombosis have all been found to increase the risk of VTE (5). Among women, additional risk factors for VTE include use of combined hormonal contraceptives (6). hormone therapy, pregnancy, and the postpartum period (7).

In Saudi Arabia, pregnancy poses an even greater risk than surgery, hospitalization, and combined hormonal contraceptive use (8), and the risk of VTE during the postpartum period are about fivefold higher than the risk during pregnancy (7). The overall incidence of pregnancy-associated VTE is currently about 200 per 100,000 women per year (9). According to the Centers for Disease Control (CDC) pregnancy-related mortality surveillance, PE was the leading cause of pregnancy-related deaths at 20%, outnumbering other complications such as hemorrhage, infections, and pregnancy-induced hypertension (10). Additionally, prior superficial vein thrombosis was found to be an independent risk factor during pregnancy or postpartum (11).

The risk of thrombosis is attributed to the homeostatic changes that occur during pregnancy. There is an increase in the concentration of clotting factors, namely, fibrinogen, von Willebrand factor, and factors VII, VIII, IX, X, and XII; which results in a hypercoagulable state. This is intended to protect pregnant women against hemorrhage, simultaneously exposing them to the potential risk of thrombosis (12). In addition to hypercoagulability, several anatomical changes occur during pregnancy: the venous stasis created by the external venous compression due to the growing uterus compromises venous outflow, subsequently increasing the susceptibility to developing thromboembolism in pregnant and postpartum women (13). Although these changes take place during pregnancy and create a higher risk of VTE during it, they also play a key role during the postpartum period (9, 13, 14).

Moreover, pregnancy combined with either heritable or acquired forms of thrombophilia results in the cumulative risk of thrombosis. A meta-analysis of pregnancy-associated thrombophilia concluded that pregnant women with heterozygous factor V Leiden mutation and prothrombin G20210A mutation had an eightfold and sevenfold increase in thrombosis risk, respectively (15). Due to the lack of prior research on pregnancy-induced thrombosis in Saudi Arabia, we conducted this study to describe the predictors of pregnancy-induced VTE (DVT and PE).

## Materials and methods

### Study design

We carried out a case-control study of patients with objectively confirmed VTE (DVT, PE, or both), induced during pregnancy or postpartum, visiting the thrombosis clinics at a major tertiary care hospital in Riyadh.

### Study participants

#### Cases

Patients who experienced one or more episodes of objectively confirmed VTE (*proximal* DVT or PE or both) during pregnancy or the postpartum period were included. We excluded intra-abdominal (splanchnic) vein, renal, gonadal, and cerebral venous thrombosis, which are often called venous thrombosis of the unusual site due to their rarity and difference in their management strategies (type and duration of anticoagulant therapy). In addition, patients with missing medical records or those with normal diagnostic imaging were excluded from this study. DVT was objectively confirmed by Doppler ultrasound and PE was diagnosed through Ventilation-perfusion (VQ) scan or CT Pulmonary Angiography (CTPA) scan. The study population, including those who developed previous episodes of single VTE, were not taking any anticoagulant therapy before the diagnosis of recurrent VTE.

#### Controls

A random sample of pregnant women attending the thrombosis clinics who did not develop VTE was selected from high-risk pregnancies. High-risk pregnancies consisted of any chronic medical condition that affected either the pregnant woman or the fetus or both, including diabetes mellitus, hypertension, chronic kidney disease, cardiac disease, inflammatory bowel disease, cancer, multiple sclerosis, and epilepsy. The ratio of controls to cases was ~1:1.

### Demographics and risk factors

Data was collected in a specifically designed strategy by reviewing the chart of the included patients. Demographic information collected included: age, weight, height, BMI before pregnancy, first-degree relative family history of VTE (venous and/or arterial thrombotic event), previous history of combined hormonal contraceptive use, and pregnancy trimester at the time of VTE diagnosis.

Data on the risk factors of VTE were collected using the risk assessment tool of the Royal College of Obstetricians and Gynecologists (RCOG) (16). Information on the risk factors collected included: C-section and pre-term delivery, postpartum hemorrhage (PPH) or blood transfusion, age, history of VTE, thrombophilia^1^, antiphospholipid syndrome (APLS), medical comorbidities, diabetes, hypothyroidism, hypertension (HTN), cardiac and lung disease, systemic lupus erythematosus (SLE), nephrotic syndrome, surgical history, BMI, parity, gross varicose veins [defined as symptomatic or above the knee or with associated phlebitis, edema/skin changes as per the RCOG guidelines (16)], current systemic infection, immobility, immobility type, hospitalization for non-delivery reasons, preeclampsia, dehydration or ovarian hyperstimulation syndrome (OHSS), recurrent abortions, and multiple pregnancies or assisted reproductive therapy (ART).

### Sample size estimate

Sample size calculations were performed using the Epi Info^TM^ program (version 7.2.5 Nov. 2021) that is provided by the Centers for Disease Control and Prevention (CDC). Accordingly, to achieve a 95% confidence interval to detect a similar odds ratio with a 5% margin of error, the sample size calculations showed that the minimum required size for the current study was 348 subjects (174 subjects per group).

### Statistical analysis

Data were analyzed using the Statistical Package for Social Sciences (SPSS) version 23. Descriptive statistics were carried out by reporting the number and percent for categorical variables, whereas continuous variables were presented as mean and standard deviations (SD). The associations between different categorical variables and VTE and continuous variables and VTE were determined through the chi-square test and student's t-test, respectively. To identify significant predictors of VTE, backward multiple logistic regression (Wald test) analyses were carried out by including all the patients' characteristics and excluding independent variables based on the probability of the Wald statistic. Only variables that showed statistical significance were reported in the results.

## Results

The current study was conducted among 180 patients diagnosed with pregnancy-associated thrombosis out of a total of 800 VTE cases in our thrombosis clinic registry; 135 (75%) of patients developed DVT, 30 (16.7%) patients developed PE, and 15 (8.3%) developed DVT that progressed to PE. Moreover, a sample of 200 subjects who did not develop VTE was included as controls.

The average ages of the patients in the control and case groups were 29.09 (±5.16) and 29.67 (±6.00) years, respectively, showing no statistically significant difference (*P* = 0.31). Similarly, no significant difference was observed in the average BMIs between the two groups, which were 31.76 (±6.48) kg/cm^2^ and 30.93 (±5.25) kg/cm^2^ in the case and control groups, respectively (*P* = 0.17). As for family history, there was a significant association between pregnancy and VTE [19.4% of cases had a family history of VTE as compared to 0.5% of the controls (*P* < 0.0001)]. In the case and control groups, 60.6 and 58.5% of the subjects were in their postpartum period, respectively. The remaining 39.4 and 41.5% of the subjects in the case and control groups were pregnant, respectively. VTE cases were almost equally distributed with a slight surge toward the first and third trimesters (13.3 and 12.8%, respectively) (Table 1).

**Table 1:** Association between baseline characteristics and VTE in cases and controls.

		**Group**		***p***
		**Case**	**Control**	
		***N*** **= 180**	***N*** **= 200**	
Age (year)	Mean (SD)	29.67 (6.00)	29.09 (5.16)	0.31
	≤ 35	149 (82.8%)	174 (87.0%)	0.25
Nationality	Saudi	180 (100.0%)	194 (97.0%)	**0.03**
	No Saudi	0 (0.0%)	6 (3.0%)	
BMI	Mean (SD)	31.76 (6.48)	30.93 (5.25)	0.17
Family history	Yes	35 (19.4%)	1 (0.5%)	**<0.001**
Pregnancy period	Postnatal	109 (60.6%)	117 (58.5%)	Reference
	First trimester	24 (13.3%)	7 (3.5%)	0.003
	Second trimester	18 (10.0%)	35 (17.5%)	0.060
	Third trimester	23 (12.8%)	41 (20.5%)	0.081
	Unknown trimester	6 (3.3%)	–	–
Surgery	Yes	3 (1.7%)	1 (0.5%)	0.35

Significant variables are in bold.

VTE was significantly associated with C- section delivery (*P* = 0.03), single previous VTE (*P* < 0.0001), antenatal previous recurrent >1 VTE (*P* < 0.0001), thrombophilia (*P* < 0.0001), APLS (*P* = 0.01), gross varicose veins (*P* < 0.0001), immobility (*P* = 0.001), hospitalization for non-delivery reasons (*P* = 0.003), and multiple pregnancy or ART (*P* = 0.03) (Table 2).

**Table 2:** Association between different risk factors and VTE in cases and controls.

**Risk factor**	**Case**	**Control**	***p***	**Unadjusted OR (95 % CI)**
	***N*** **= 180**	***N*** **= 200**		
Antenatal period	71 (39.4%)	82 (41.0%)	0.76	1.07 (0.71–1.61)
Postnatal period	109 (60.6%)	118 (59.0%)		
C-section delivery	52 (47.7%)	40 (33.9%)	**0.03**	**1.78 (1.04–3.04)**
Preterm delivery	7 (6.4%)	4 (3.4%)	0.29	1.96 (0.56–6.88)
PPH or blood transfusion	5 (4.6%)	1 (0.8%)	0.10	5.72 (0.66–49.76)
Family history	35 (19.4%)	1 (0.5%)	**<0.0001**	**48.03 (6.51–354.66)**
Age >35	31 (17.2%)	26 (13.0%)	0.25	1.39 (0.76–2.54)
Single previous VTE	33 (18.3%)	4 (2.0%)	**<0.0001**	**11.00 (3.81–31.73)**
Antenatal previous recurrent > 1 VTE	13 (7.2%)	0 (0.0%)	**<0.0001**	NA
Thrombophilia	21 (11.7%)	1 (0.5%)	**<0.0001**	**26.28 (3.50–197.51)**
APLS	10 (5.6%)	2 (1.0%)	**0.01**	**5.82 (1.26–26.95)**
Medical comorbidities	56 (31.1%)	56 (28.0%)	0.51	1.16 (0.75–1.82)
Diabetes	7 (5.1%)	12 (8.1%)	0.32	0.61 (0.23–1.61)
Hypothyroidism	19 (13.8%)	27 (18.0%)	0.33	0.73 (0.38–1.38)
Hypertension	5 (3.6%)	5 (3.3%)	1.00	1.09 (0.31–3.85)
Cardiac disease	1 (0.7%)	0 (0.0%)	0.48	NA
Lung disease	1 (0.7%)	0 (0.0%)	0.48	NA
SLE	7 (5.1%)	2 (1.3%)	0.09	3.95 (0.81–19.37)
Nephrotic syndrome	1 (0.7%)	0 (0.0%)	0.48	NA
Surgical procedures	3 (1.7%)	3 (1.5%)	1.00	1.11 (0.22–5.59)
Obesity (BMI >30)	73 (40.6%)	93 (46.5%)	0.24	0.78 (0.52–1.18)
Parity ≥3	58 (32.2%)	56 (28.0%)	0.37	1.22 (0.79–1.90)
Gross varicose veins	26 (14.4%)	2 (1.0%)	**<0.0001**	**16.71 (3.91–71.51)**
Current systemic infection	4 (2.2%)	6 (3.0%)	0.75	0.73 (0.20–2.65)
Immobility	9 (5.0%)	0 (0.0%)	**0.001**	NA
Immobility type			NA	NA
Paraplegia	0 (0.0%)	0 (0.0%)		
Long distance	8 (88.9%)	0 (0.0%)		
Travel > 6 h				
PGP	0 (0.0%)	0 (0.0%)		
Other	1 (11.1%)	0 (0.0%)		
Hospitalization for non-delivery reasons	12 (6.7%)	2 (1.0%)	**0.003**	**7.07 (1.56–32.04)**
Preeclampsia	8 (4.5%)	3 (1.5%)	0.09	3.07 (0.80–11.76)
Dehydration or OHSS	3 (1.7%)	0 (0.0%)	0.10	NA
Recurrent abortions	25 (13.9%)	24 (12.0%)	0.58	1.18 (0.65–2.16)
Multiple pregnancy or ART	17 (9.4%)	8 (4.0%)	**0.03**	**2.50 (1.05–5.95)**

VTE, Venous thromboembolism; PPH, Postpartum hemorrhage; APLS, Antiphospholipid syndrome; SLE, Systemic lupus erythematosus; PGP, Pelvic girdle pain; OHSS, Hyperemesis ovarian hyperstimulation syndrome; ART, Assisted reproductive technology. Significant variables are in bold.

**Figure 1:**
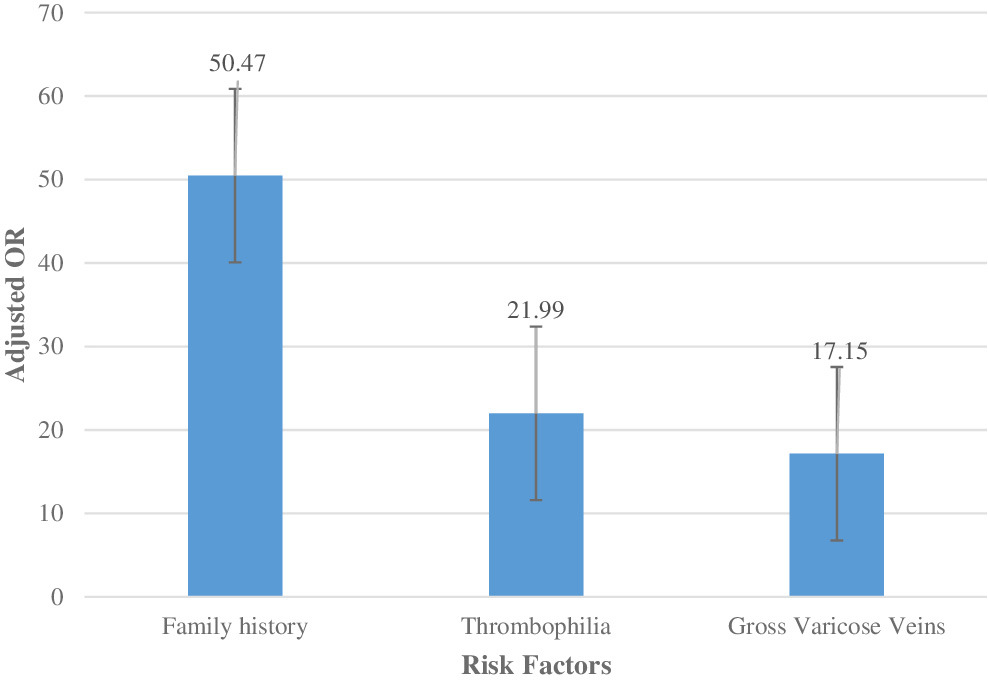
Adjusted ORs for each significant factor of VTE in the multiple logistic regression model.

After entering all the significant variables from the univariate analysis into multivariate analysis, the only variables that remained significant were family history (adjusted OR = 50.47, 95% CI: 6.78–375.64, *P* < 0.0001), thrombophilia (adjusted OR = 21.99, 95% CI: 2.83–170.63, *P* = 0.003), and gross varicose veins (adjusted OR = 17.15, 95% CI: 3.93–74.87, *P* < 0.0001). Thus, VTE is more likely to develop 50.47 times, 21.99 times and 17.15 times in cases with family history, thrombophilia, and gross varicose veins, respectively (Figure 1). We noticed that the adjusted ORs were almost similar to unadjusted ORs. This could probably be due to potential confounders like other unmeasured factors that could not be determined due to data limitations.

## Discussion

The results of the present study show that family history increases the risk of developing VTE. These findings are consistent with the findings from a case-control study carried out by Gader et al. (17) among Sudanese pregnant and postpartum patients with VTE (OR: 7.4). Correspondingly, Bezemer et al. (18) and Zoller et al. (19) also reported that a positive family history of VTE increases its risk twofold to fourfold, depending on the number of affected relatives.

Similarly to our results, the significant association of VTE with thrombophilia was also reported by James et al. The latter found that, in pregnant women with thrombophilic abnormalities, the risk of VTE was estimated to be 51.8-fold and 15.8-fold in those with hereditary thrombophilia and APLS, respectively (20). A possible explanation for thrombophilia is the effect of blood abnormalities on the levels of coagulation factors and other circulating blood proteins (participating in the coagulation cascade) (21).

Thrombophilia is defined as the disruption in the balance of “procoagulant” and “anticoagulant” activity, which determines the likelihood of thrombosis in a patient (22). A univariate analysis indicated that recurrent VTE was significantly associated with thrombosis. However, similar results were not observed after a multivariate analysis, which was congruent with previous studies (23, 24). A study conducted by Middeldorp among non-pregnant patients also concluded that no association was found between thrombosis abnormalities and VTE (24). This could be explained by the global increase of thrombotic risk in pregnant women and the synergistic interaction with inherited thrombophilia, allowing for the demonstration of an association that is not statistically significant in the general population. Therefore, inherited thrombophilia seems to be the major cause of adverse pregnancy outcomes including fetal loss, preeclampsia, abruptions, severe intrauterine growth restriction, and early onset (9, 25–27). The most frequent clinically significant inherited thrombophilias are Factor V Leiden and Factor II (prothrombin) G20210A followed by deficiencies in proteins C, S, and antithrombin; dysfibrinogenemia, and hyperhomocysteinemia (15, 25–30). However, previous studies demonstrated a weaker association between inherited thrombophilia [Factor V Leiden and Factor II (prothrombin) G20210A] and pregnancy-associated VTE risk (6). Correspondingly, some studies suggested that inherited thrombophilia had some impact on adverse pregnancy outcomes, suggesting that it is contributory instead of a major cause (25, 29–31). Furthermore, the effect of the changes in hemostatic, fibrinolytic, and anticoagulant proteins during pregnancy expose pregnant women to an increased risk of thromboembolism and, therefore, aggravate the effects of inherited thrombophilia's (9, 15, 26).

The last risk factor found to be significantly associated with VTE was gross varicose veins. Similar to our observations, a significant association between varicose veins and DVT was observed among a general practice population with documented varicose veins, in a study carried out by Muller-Buhl et al., in a primary care center in Heidelberg, Germany. They reported that there were 132 DVT episodes among 2,357 patients with varicose veins (5.6 %) compared to 728 out of 80,588 patients of the cohort without varicose veins (0.9 %) (*P* < 0.0001) (32). This can be explained by the fact that varicose veins cause chronic venous insufficiency and thus becomes a possible risk factor for DVT (33). On the other hand, it was reported by Heit et al. (34) that the risk of DVT imparted by varicose veins is uncertain and appears to vary with the patient's age.

It is important to note that both the case and control groups consisted of pregnant women from the high-risk group. As their characteristics and prognosis were similar, no other factors would have impeded the results. Additionally, compression ultrasound sonography (CUS) was performed in all patients as the first line for diagnosis of PE (35). If the results of CUS were negative further investigating would be used such as confirmatory diagnostic imaging for PE, to minimize radiation exposure to the patients (36).

Therefore, it is recommended that a formal, written risk assessment of VTE risk factors be performed before pregnancy, at the time of antenatal booking, and at the time of delivery to reduce VTE during pregnancy. Given the ongoing deliberation to introduce universal screening for thrombophilia before pregnancy, this study could play an essential role in helping obstetricians to decide on the use of a prophylactic treatment such as anticoagulant therapy. It is therefore also recommended that obstetricians refer women with gross varicose veins planning for pregnancy to vascular surgeons for proper management of varicose veins before pregnancy where any intervention would be delayed if possible. Additionally, obstetricians are recommended to screen pregnant women for VTE risk factors addressed by RCOG, particularly varicose veins and those with a family history of VTE to help them make more time-sensitive decisions regarding the use of anticoagulant thromboprophylaxis during their patient's pregnancy supported by a multi-disciplinary discussion.

Moreover, prophylaxis is advised for women who are at high risk of pregnancy-associated VTE, such as those with inherited thrombophilias, a strong family or personal history of VTE, and those with gross varicose veins.

The results of this study should be evaluated in light of its strengths and limitations. Being one of the few studies, addressing this important topic in the Middle East is the main strength of our study. The critical limitation of our study was the retrospective design, where potential confounding by other unmeasured factors could not be taken into account due to data limitations.

## Conclusion

In summary, the findings of this study show that family history, thrombophilia, and gross varicose veins were the provoking factors for VTE, exceeding other transient risk factors during pregnancy and the postpartum period. Further larger studies using a randomized design need to be conducted to confirm the results of our study and to identify a more predictive risk factor during pregnancy and the postpartum period. Recommendations for including a multidisciplinary team approach to the management of pregnant women or those seeking pregnancies taking into account the VTE risk factors is highly encouraged.

## Data availability statement

The original contributions presented in the study are included in the article/supplementary material, further inquiries can be directed to the corresponding author.

## Ethics statement

The studies involving human participants were reviewed and approved by KFMC IRB. The patients/participants provided their written informed consent to participate in this study.

## Author contributions

MAA carried out the study, participated in the study design, and wrote the final manuscript. AA conceived the study and participated in its design and in drafting of the manuscript. RAA participated in the study design, interpretation of data, and drafting of the manuscript. RMA contributed to the design of the study, managed the literature search, and drafted the manuscript. AZ participated in the interpretation of data and drafting of the article. OA, MA, and AA-S participated in the study design, interpretation of data, and drafting of the manuscript. All authors read and approved the manuscript.

## Conflict of interest

The authors declare that the research was conducted in the absence of any commercial or financial relationships that could be construed as a potential conflict of interest.

## Publisher's note

All claims expressed in this article are solely those of the authors and do not necessarily represent those of their affiliated organizations, or those of the publisher, the editors and the reviewers. Any product that may be evaluated in this article, or claim that may be made by its manufacturer, is not guaranteed or endorsed by the publisher.

## Footnotes

1 Thrombophilia is a group of inherited or acquired disorders that increase the risk of venous or arterial thrombosis. Hereditary thrombophilia is due to antithrombin deficiency, protein C or protein S deficiency, factor V Leiden mutation, and prothrombin gene mutation. Acquired thrombophilia is frequently associated with antiphospholipid syndrome and, activated protein C resistance; which is characterized by thrombosis and the presence of lupus anticoagulant, anticardiolipin, and anti-beta2-glycoprotein antibodies.
